# Fine-Tuning and Optimization of Superconducting Quantum Magnetic Sensors by Thermal Annealing

**DOI:** 10.3390/s19173635

**Published:** 2019-08-21

**Authors:** Antonio Vettoliere, Berardo Ruggiero, Massimo Valentino, Paolo Silvestrini, Carmine Granata

**Affiliations:** 1 Institute of Applied Sciences and Intelligent Systems of National Research Council (CNR), 80078 Pozzuoli, Italy; 2 Department of Mathematics and Physics, University of Campania “L. Vanvitelli”, 81100 Caserta, Italy

**Keywords:** magnetic sensors, SQUIDs, magnetic field noise, annealing

## Abstract

In the present article, we present the experimental results concerning the fine-tuning and optimization of superconducting quantum interference device (SQUID) parameters by thermal annealing. This treatment allows for the modification of the parameters in order to meet a specific application or to adjust the device parameters to prevent the increase of magnetic field noise and work instability conditions due to a different critical current with respect to the design value. In particular, we report the sensor critical current, the voltage–flux (V–Φ) characteristics and the spectral density of the magnetic field of SQUID magnetometers for different annealing temperatures. The measurements demonstrate that it is possible to achieve a fine control of the most important device parameters. In particular, we show that thermal annealing allows for the reduction of SQUID noise by more than a factor of 5 and makes the device working operations very stable. These results are very useful in view of quantum technology applications related to superconducting quantum computing where the correct functioning of the quantum bit depends on the fine control of the superconducting quantum device parameters and selectable annealing is possible by using a suitable laser as a thermal source.

## 1. Introduction

Quantum sensing is an intriguing and interesting topic in applied physics. In fact, many efforts are devoted to the development of quantum technology in view of challenging applications such as quantum information and computing and ultra-high sensitivity sensors able to investigate the matter at a nanoscale level [1,2].

Since the first superconducting quantum interference devices (SQUIDs) were developed over 50 years ago, their application potential has stimulated continuous scientific and technological research on these quantum sensors. SQUIDs remain among the most sensitive magnetic sensors that are, practically, irreplaceable in all applications that require extreme sensitivity. Such applications include basic physics, geophysics, nanomagnetism, biomedicine, and quantum computing [3,4,5]. Among the many applications of SQUIDs, the most important are the well-established magnetoencephalography (MEG) [6,7,8,9] and the promising applications in the field of quantum computation, where SQUIDs are employed as flux quantum bits (qubits). In both applications, it is fundamental to have very good control of the most important characteristics of the SQUID device, as well as good reliability and robustness. In fact, in the flux qubit, which is one of the three superconducting qubits, the circuit is designed to give a double-well potential, with a height depending on the critical current of the Josephson elements [10,11,12].

The core of a SQUID device is a superconducting ring interrupted by two Josephson junctions. Due to quantum interference, the phase difference of the two junctions is related to the flux linkage with the superconducting ring normalized to the elementary flux quantum (Φ_0_ = 2.07 × 10^−15^ Wb). The most important features of a SQUID device depend on the following parameter: Φ_L_ = LI_0_/Φ_0,_ where I_0_ is the SQUID critical current and L is the ring inductance. Since the inductance essentially depends on the geometric characteristics of the loop and therefore on the SQUID design, the possibility to control the critical current allows us to modify the most important characteristics of a SQUID device, such as the modulation depth, the voltage swing, and the noise spectral density of magnetic flux, as well as the double-well potential in the case of radiofrequency (rf) SQUIDs [3]. These applications require a large number of SQUID sensors having ultra-high sensitivity and good working stability.

In this paper, we report an experimental study on the fine control and optimization of the main SQUID magnetometer parameter. In particular, by using thermal annealing, we show that we are able to modify the critical current of SQUID magnetometers of about one order of magnitude with a step of a few percent of the initial critical current. As we show, this allows for the optimization of the main characteristics of the quantum sensor, such as the voltage-flux characteristics and the spectral density of the magnetic flux and field noise.

## 2. Thermal Annealing Procedure

The Josephson current in a SQUID is a tunnel current of Cooper pairs due to an overlap of the macroscopic wave function relative to the superconductors of the Josephson junction. The maximum Josephson current value, known as the Josephson critical current, depends on the thickness of the insulator barrier. In particular, the Josephson critical current density follows an exponential law: *J_C_* = *J*_*C*,0_ *e*^−*At*^, where *A* is a coefficient depending on the superconductor and insulator materials and *t* is the thickness of the insulator barrier [13,14]. Therefore, a small variation of the barrier thickness causes an appreciable change of the critical current density. For this reason, the critical current value, especially in the fabrication of a large number of Josephson devices, can be different with respect to the design value. Thermal annealing allows increasing in a reliable and fine way the thickness of the insulator barrier.

In the case of all-refractory niobium technology, the insulation barrier consists of a thin layer of aluminum oxide (AlOx) typically obtained by the thermal oxidation of the aluminum film in a controlled oxygen atmosphere. In our case, the vacuum chamber was filled with dry oxygen at a pressure of 250 mbar for 1 h. In this condition, we obtained a critical current density of about 70 A/cm^2^ [15].

It is well known that a change of the Josephson critical density can be obtained by thermal annealing [16,17,18,19]. In fact, during this process, the thickness of the tunnel barrier (AlOx) can increase due to the migration of reacting oxygen coming from the niobium oxide surface through the channels formed in the niobium granular structure. Also, unbound oxygen embedded at interstitial places close to the tunneling barrier can react with aluminum atoms, increasing the insulation layer.

If well controlled, thermal annealing can be employed to tune the critical current of both the Josephson junction and the SQUID magnetometer. In our experimental study, we employed a programmable hot plate (CEE 1100-Brewer Science Inc., Rolla, Missouri, USA) reaching a maximum temperature of 280 °C with 0.1 °C resolution. The hot plate was used in hard-contact mode, where a suitable vacuum guaranteed an effective and uniform annealing. An alternative way to obtain a thermal annealing is to use a laser that allows you to perform selectable annealing with a high spatial resolution (a few µm), though the heating temperature is more difficult to control [20,21].

The annealing and the characterization were performed on fully integrated SQUID magnetometers with a design based on a Ketchen-type scheme. Since the design and the fabrication of the SQUID magnetometers is well described elsewhere, only a short description is reported here. The SQUID magnetometer consists of a single square-coil pickup coil (A_p_ = 64 mm^2^) connected in series with a multiturn input coil which is magnetically coupled to the SQUID loop in a washer configuration. An integrated feedback coil in bipolar configuration to reduce the cross-talk effect and an additional positive feedback (APF) circuit consisting of both a square coil surrounding the pickup coil and a resistor network are integrated in the same chip containing the magnetometer [22].

The SQUID model used for the design optimization was a standard model based on resistively shunted junctions where the white noise is essentially due to the Johnson–Nyquist noise of the resistors in parallel with the Josephson junctions [1,2].

It is worth noting that the results reported below are also valid for different SQUID magnetometer designs and in general for any devices including Josephson tunnel junctions, while they are not applicable for other types of weak links, such as Dayem bridges or contact points.

**Figure 1:**
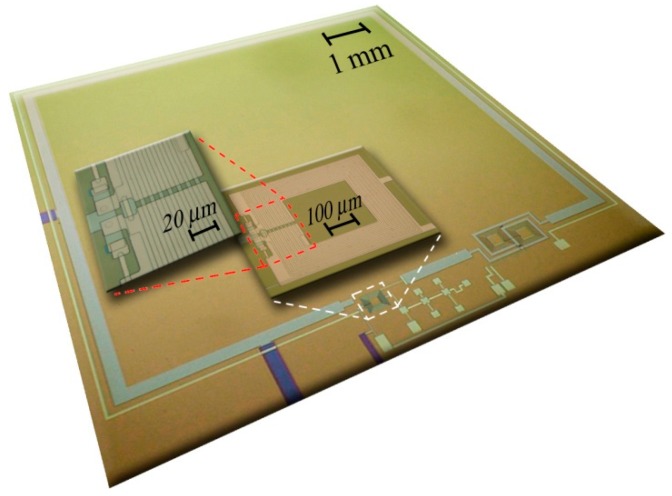
Fully integrated superconducting quantum interference device (SQUID) magnetometers including a bipolar coil for flux-locked-loop operation and an additional positive feedback circuit consisting of a narrow square coil (light green) surrounding the magnetometer pickup coil (light blue) and a network resistor.

The SQUID magnetometer fabrication is based on all-refractory niobium technology (Figure 1). The Nb/Al-AlOx/Nb trilayer was made by depositing thin films by direct current (dc) magnetron sputtering in an ultra-high vacuum station without vacuum breaking. The window-type Josephson junctions (4 × 4 µm^2^) were realized by using standard photolithography and a selective niobium anodization process (SNAP).

## 3. Experimental Results

The characterization of SQUID magnetometers was performed at 4.2 K, with the SQUID immersed in liquid helium inside a twin cylindrical shield made of lead and µ-metal [23,24]. All the electrical connections to the room temperature electronics were radiofrequency-filtered. The white magnetic flux noise was measured using a very low-noise readout electronics based on flux-locked-loop (FLL) with a direct-coupled scheme. In such a configuration, the SQUID is directly coupled to a low-noise preamplifier. The FLL linearizes the SQUID output, increasing the linear dynamic range. The whole amplification stage is integrated on a miniaturized detection circuit and carefully shielded by a copper box. After each annealing step, a full magnetometer characterization consisting of the measurements of the critical current, voltage–magnetic flux characteristic, spectral density of the magnetic field, and flux noise was carried out.

**Figure 2:**
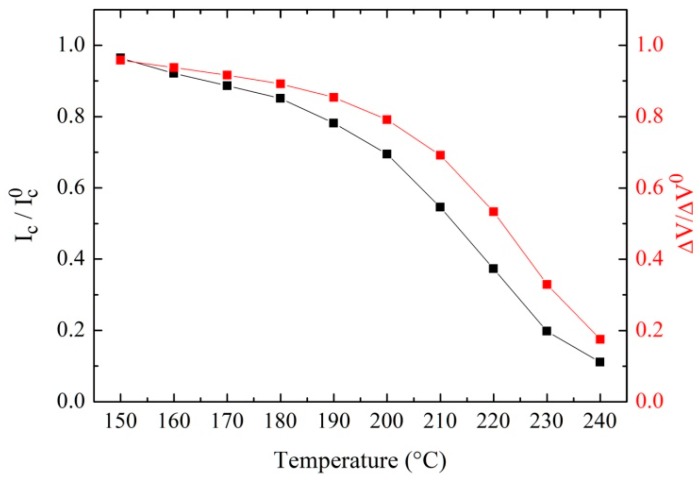
Critical current and voltage swing of a SQUID magnetometer as a function of the annealing temperature measured at liquid helium temperature. The values were normalized to the initial value without any annealing. The annealing time for each temperature was 30 min. The solid black line is just an eye-guide line.

Figure 2 reports the critical current and the voltage swing as a function of the annealing temperature ranging from 150 to 240 °C. The current and voltage values were normalized to those with no annealing, while the bare values are reported in Table 1. Each annealing step took 30 min, during which the magnetometer was heated without the chip carrier and anchored on the hot plate by vacuum. For both I_C_ and ΔV, we can observe a variation of about one order of magnitude with respect to the initial values. Both curves show a slow variation for lower temperatures, while above 180 °C the values decrease at a faster rate. The same behavior has been observed for different SQUID magnetometers. It is therefore conceivable that the oxygen migration process toward the barrier is not linear with the annealing temperature. In Figure 2, we report the variation for a step of 10 °C.

However, we stress that it is possible to have a much smaller variation of the critical current by decreasing the temperature steps. In particular, in the range of 180–240 °C, we were able to induce in a very reliable way a critical current variation as low as 2–3% for a temperature step of 2 °C. Since, in both dc and rf SQUID the inductance parameter β_L_ depends linearly on the critical current, this procedure enabled us to modify the β_L_ of a SQUID magnetometer by about a factor of 10.

**Figure 3:**
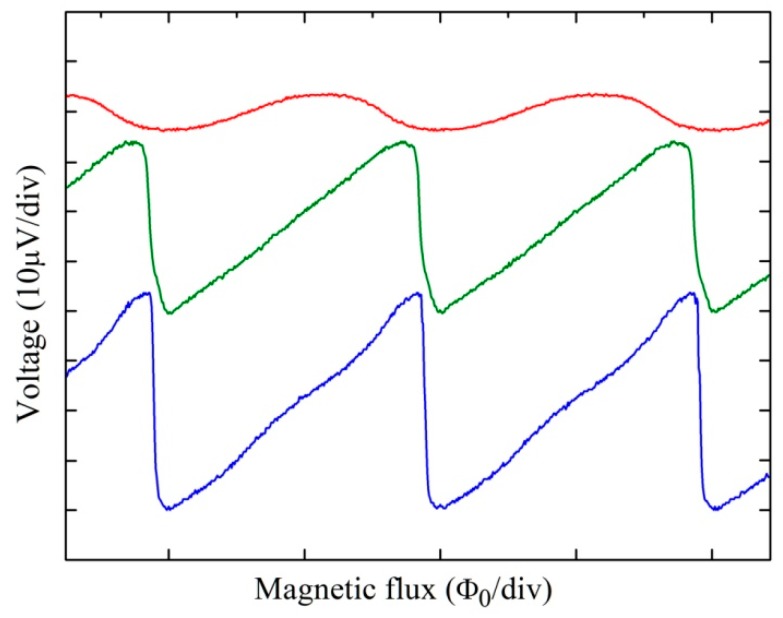
SQUID voltage as a function of the external magnetic flux measured at T = 4.2 K (lower curve). The medium (green) and the top (red) traces refer to the same magnetometer for an annealing temperature of 200 and 240 °C, respectively.

In Figure 3, we report the voltage–magnetic flux characteristics measured at liquid helium temperature of the SQUID magnetometer under investigation. The green (middle) curve depicts the characteristic where the overall annealing process produced the best magnetic field noise (T = 200 °C), while the blue (lower) and the red (top) curves are relative to the untreated sample and to the highest annealing temperature (T = 240 °C), respectively. In order to verify the possibility of modifying in a wide range the parameter of the SQUID magnetometers, we chose a sample with a high critical current showing a peaked V–Φ characteristic and a high magnetic noise level. As it is evident from Figure 3, the lower V–Φ curve shows a very sharp side due to the high critical current. In fact, the voltage responsivity (V_Φ_ = ∂V/∂ Φ_e_) gain is given by [25,26]:

(1) $$\frac{V_{\Phi }^{A}}{V_{\Phi }^{\mathrm{int}}}=\frac{1}{1-G_{A}};\;G_{A}=V_{\Phi }^{\mathrm{int}}\frac{M_{A}}{R_{A}};\;V_{\Phi }^{\mathrm{int}}≅\frac{I_{C}\;R}{\left(1+\beta_{L}\right)\;\Phi_{0}}$$

where G_A_ is the APF gain, M_A_ is the mutual inductance between the SQUID loop and the APF coil, and R_A_ is the resistance of the APF circuit. If the critical current is rather high, the intrinsic V_Φ_ increases and, consequently, G_A_ increases. If G_A_ approaches the unit value, the APF circuit becomes unstable and a hysteresis appears in the V–Φ characteristic.

Since the thermal annealing decreases the critical current, it is possible to reduce the G_A_ to smooth the V–Φ characteristic, as shown in the middle (green) curve in Figure 3. In this case, the I_C_ = 34.5 µA corresponds to about 70% of the initial value (42.3 µA). It is worth noting that the V–Φ characteristic is now smooth, stable, and preserves a suitable responsivity gain. Of course, if the critical current decreases excessively, as in the case of the top (red) curve of Figure 3, the voltage swing and the intrinsic responsivity decrease up to the point of a degradation of magnetometer performance. In this case, the decreases of I_C_ were so high as to reach 10% of the initial value.

**Figure 4:**
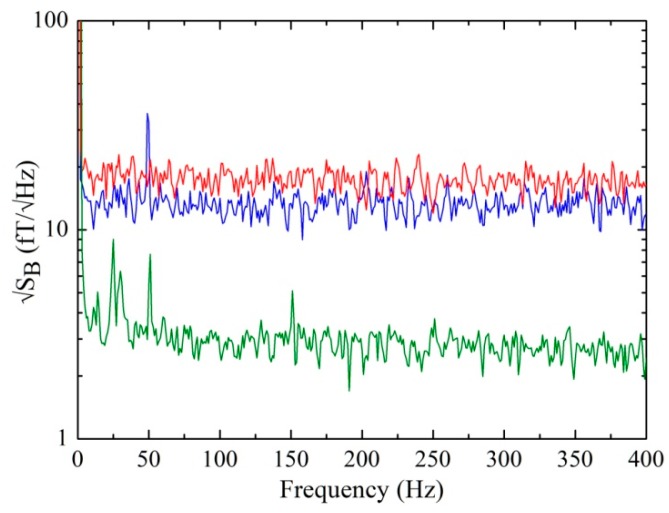
Spectral densities of the magnetic noise of a SQUID magnetometer. The lower (green) and top (red) spectra refer to an annealing temperature of 200 and 240 °C, respectively, while the medium (blue) spectrum corresponds to the device without annealing.

Figure 4 reports the spectral densities of the field noise measured in the flux-locked-loop mode at T = 4.2 K, corresponding to the three V–Φ characteristics reported in Figure 3. Due to the flicker noise at low frequency with a *1/f* corner of about 3 Hz, the magnetic field noise values at 1 Hz are about 3 times the white noise values. Note that the magnetic noise of the magnetometer without any annealing (blue curve) is more than 4 times greater than that after the optimum annealing treatment, demonstrating full effectiveness in tuning and optimizing SQUID magnetometer parameters.

In fact, the annealing procedure causes a progressive and controlled reduction of the Josephson critical current up to the optimal value, leading to a β_L_ equal to about 1 and thus guaranteeing noise optimization as predicted by SQUID theory [2,3]. In addition, the critical current decrease leads to a reduction of the additional positive feedback circuit gain, ensuring good work stability [25,26].

As expected, the magnetic noise corresponding to the smallest V–Φ characteristic is rather high because the voltage swing and the intrinsic responsivity are very low and the critical current is far from the value that optimizes the β_L_ parameter.

From a practical point of view, it should be emphasized that the noise of 13 fT/Hz^1/2^ shown by the SQUID magnetometer prevents MEG applications, while the same magnetometer after the annealing process can be applied in all high-sensitivity applications, including MEG.

**Figure 5:**
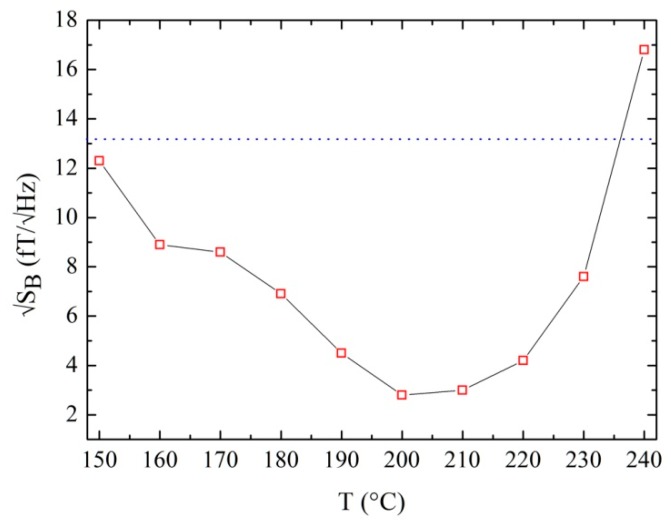
White spectral density of magnetic field noise as a function of the annealing temperature measured a T = 4.2 k in flux-locked-loop mode. The solid black line is just an eye-guide line.

In Figure 5, we report the white values of the magnetic field noise as a function of the annealing temperature. The horizontal dotted line indicates the white magnetic noise before the annealing. As we can see from the figure, the noise decreases for annealing temperatures up to 200 °C, after which it increases, and for T = 240 °C it exceeds the initial value. This behavior at the first stage is due to the decrease of G_A_ that reduces hysteresis and the instability device, and, as a consequence, the noise. By decreasing the critical current further, a decrease of intrinsic responsivity occurs and, at some point, it causes a reversal of the noise trend.

**Figure 6:**
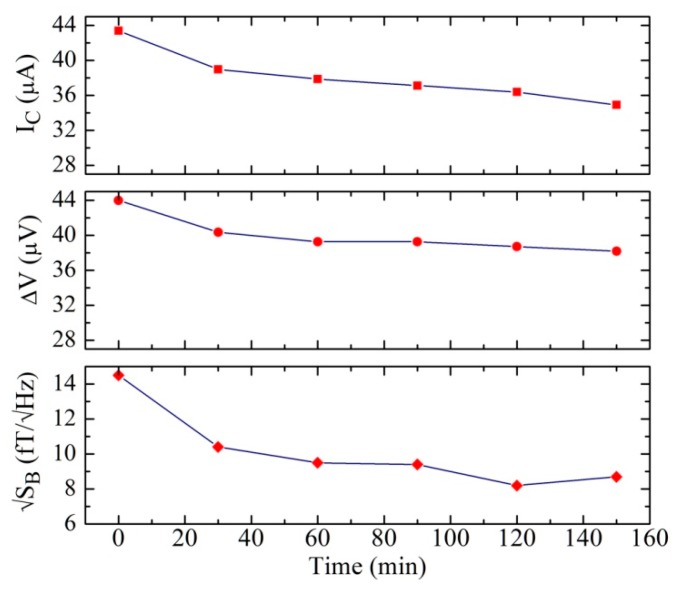
Critical current (top), voltage swing (middle), and white magnetic field noise of a SQUID magnetometer as a function of the annealing time for a fixed annealing temperature (T_a_ = 170 °C).

In order to also obtain information on the temporal dependence for a fixed temperature of the annealing process, we made a complete characterization of another SQUID magnetometer by fixing the annealing temperature and varying the time. In particular, we chose an annealing temperature of 170 °C and increased the time with a step of 30 min. Figure 6 reports the critical current, the voltage swing, and the white spectral density of the magnetic field noise measured at T = 4.2 K. From Figure 6, it is evident that there are slight variations as a function of the time. In particular, after 150 min of annealing, we measured a critical decrease of about 20% and even less for the voltage swing. 

A greater variation can be observed for the magnetic field noise, because even small decreases of the critical current can have a significant effect on the APF gain and the stability of the SQUID magnetometer. The same measurements were also made for different annealing temperatures, and we observed the same behavior. Due to the observed slight variations, this procedure can be effectively used to fine-tune the magnetometer parameters.

## 4. Conclusion

In conclusion, an experimental study on the annealing of SQUID magnetometers has been presented. In particular, detailed measurements of main SQUID magnetometer parameters, such as the critical current, the voltage swing, and the magnetic noise as a function of the annealing temperature, have been shown. These measurements highlight the possibility of optimizing the performances and fine-tuning the most important parameters of a SQUID magnetometer. We believe that this study is very important for applications that require the optimization and fine control of the SQUID key parameters. In addition to high-sensitivity applications such as biomagnetism, it can certainly be useful for superconducting quantum computing where a fine control of the superconducting quantum device parameters is required. In the latter application, it is preferable to perform annealing with a laser, which allows for the controlled heating of the devices contained on the chip [20,21].

## Figures and Tables

**Table 1:** Main superconducting quantum interference device (SQUID) parameters for different annealing temperatures measured at liquid helium temperature. Each annealing step lasts 30 min.

T (°C)	I_c_ (µA)	ΔV (µV)	√S_B_ (fT/√Hz)
-	42.30	43.6	13.2
150	40.08	41.8	12.3
160	38.97	40.9	8.9
170	37.50	40.0	8.6
180	36.00	38.9	6.9
190	33.08	37.2	4.5
200	29.40	34.5	2.8
210	23.10	30.2	3.0
220	15.79	23.3	4.2
230	8.38	1.44	7.6
240	4.70	0.76	16.8

## References

[1] Bader S.D. (2006). Colloquium: Opportunities in nanomagnetism. Rev. Modern Phys..

[2] Granata C., Vettoliere A. (2016). Nano Superconducting Quantum Interference device: a powerful tool for nanoscale investigations. Phys. Rep..

[3] Clarke J., Braginski A.I. (2004). The SQUID Handbook Vol I: Fundamentals and Technology of SQUIDs and SQUID Systems.

[4] Seidel P. (2015). Applied Superconductivity: Handbook on Devices and Applications.

[5] Clarke J., Braginski A.I. (2006). The SQUID Handbook Vol II: Fundamentals and Technology of SQUIDs and SQUID Systems.

[6] Sternickel K., Braginski A.I. (2006). Biomagnetism using SQUIDs: Status and perspectives. Supercond. Sci. Technol..

[7] Cohen D., Halgren E. (2009). Magnetoencephalography. Encycl. Neurosci..

[8] Del Gratta C., Pizzella V., Tecchio F., Romani G.L. (2001). Magnetoencephalography—A noninvasive brain imaging method with 1 ms time resolution. Rep. Progr. Phys..

[9] Rombetto S., Granata C., Vettoliere A., Russo M. (2014). Multichannel system based on a high sensitivity superconductive sensor for magnetoencephalography. Sensors.

[10] Ladd T.D., Jelezko F., Laflamme R., Nakamura Y., Monroe C., O’Brien J.L. (2010). Quantum computers. Nature.

[11] Chiorescu I., Nakamura Y., Harmans C.J.P.M., Mooij J.E. (2003). Coherent Quantum Dynamics of a Superconducting Flux Qubit. Science.

[12] Niskanen A.O., Harrabi K., Yoshihara F., Nakamura Y., Lloyd S., Tsai J.S. (2007). Quantum Coherent Tunable Coupling of Superconducting Qubits. Science.

[13] Barone A., Paterno G. (1982). Physics and Applications of the Josephson Effect.

[14] Van Duzer T., Turner C.W. (1999). Principles of Superconductive Devices and Circuits.

[15] Granata C., Vettoliere A., Russo M. (2011). An ultralow noise current amplifier based on superconducting quantum interference device for high sensitivity applications. Rev. Sci. Instrum..

[16] Migacz J.V., Huber M.E. (2003). Thermal annealing of Nb/Al-AlO/sub x//Nb Josephson junctions. IEEE Trans. Appl. Supercond..

[17] Shiota T., Imamura T., Hasuo S. (1992). Fabrication of high quality Nb/AlO/sub/x-Al/Nb Josephson junctions. III. Annealing stability of AlO/sub/x tunneling barriers. IEEE Trans. Appl. Supercond..

[18] Oliva A., Monaco R. (1994). Annealing properties of high quality Nb/Al-AlO/sub x//Nb tunnel junctions. IEEE Trans. Appl. Supercond..

[19] Lehnert T., Billon D., Grassi C., Gundlach K.H. (1992). Thermal annealing properties of Nb-Al/AlOx-Nb tunnel junctions. J. Appl. Phys..

[20] Granata C., Vettoliere A., Petti L., Rippa M., Ruggiero B., Mormile P., Russo M. (2007). Localized laser trimming of critical current in niobium based Josephson devices. Appl. Phys. Lett..

[21] Granata C., Petti L., Rippa M., Rombetto S., Ruggiero B., Russo M., Russo R., Vettoliere A. (2013). Spatial modulation of critical current density in niobium based Josephson junctions induced by selective heating. App. Phy. Lett..

[22] Granata C., Vettoliere A., Russo M. (2007). Miniaturized superconducting quantum interference magnetometers for high sensitivity applications. Appl. Phys. Lett..

[23] Vettoliere A., Granata C., Esposito E., Russo R., Petti L., Ruggiero B., Russo M. (2009). Performance of high-sensitivity nano-SQUIDs based on niobium Dayem bridges. IEEE Trans. Appl. Supercond..

[24] Granata C., Russo R., Esposito E., Vettoliere A., Russo M., Musinu A., Peddis D., Fiorani D. (2013). Magnetic properties of iron oxide nanoparticles investigated by nanoSQUIDs. Eur. J. Phys. B.

[25] Drung D., Koch H. (1994). An integrated dc SQUID magnetometer with variable additional positive feedback. Supercond. Sci. Technol..

[26] Drung D., Weinstock H. (1996). Advanced SQUID read-out electronics. SQUID Sensors: Fundamental, Fabrication and Application.

